# Myosteatosis predicts the prognosis of patients with ST-elevation myocardial infarction who undergo emergency percutaneous coronary intervention

**DOI:** 10.3389/fendo.2025.1545706

**Published:** 2025-03-28

**Authors:** Junqian Wang, Lingshan Zhou, Yuan Yang, Yiqing Wang, Yan Liang, Tong Wang, Jinkui Li, Ming Bai

**Affiliations:** ^1^ Heart Center, The First Hospital of Lanzhou University, Lanzhou, Gansu, China; ^2^ Gansu Clinical Medical Research Center for Cardiovascular Diseases, The First Hospital of Lanzhou University, Lanzhou, Gansu, China; ^3^ Department of Geriatrics Ward 2, The First Hospital of Lanzhou University, Lanzhou, Gansu, China; ^4^ Department of Gastroenterology, The First Affiliated Hospital, Hengyang Medical School, University of South China, Hengyang, Hunan, China; ^5^ Department of Radiology, The First Hospital of Lanzhou University, Lanzhou, China

**Keywords:** myosteatosis, fat infiltration, muscle quality, sarcopenia, ST-segment elevation myocardial infarction

## Abstract

**Objective:**

To investigate the value of myosteatosis in predicting the prognosis of patients with ST-elevation myocardial infarction (STEMI).

**Methods:**

This retrospective study involved 324 patients with STEMI who had undergone emergency percutaneous coronary intervention (PCI) at our institution between 2017 and 2020. Myosteatosis was assessed using mean muscle attenuation (MMA). Cox proportional hazards models were utilized to identify prognostic determinants required for the construction of a nomogram. The discriminatory performance of the nomogram was assessed via calibration curve analysis.

**Results:**

Among the 324 patients, 35 patients (10.8%) died during the follow-up period. A lower MMA was observed in patients who died after discharge. In the multivariate analysis, MMA was identified as an independent prognostic factor. The optimal cutoff MMA value for the prediction of all-cause mortality was 32.5 Hu. The patients were classified into high (≥32.5, n=208) and low (<32.5, n=116) MMA groups. Compared with patients in the high-MMA group, patients in the low-MMA group had shorter overall survival (OS). Finally, nomograms for OS that integrate the MMA and other clinical parameters were constructed. The calibration analysis revealed that the nomograms accurately predicted the 1-, 3- and 5-year OS rates of patients.

**Conclusions:**

Myosteatosis was associated with poorer survival outcomes in STEMI patients who underwent emergency PCI. A novel risk model comminating myosteatosis with other common clinical indicators can accurately predict the prognosis of STEMI patients.

## 1 Introduction

STEMI is the most severe type of coronary heart disease. Despite continuous optimization of primary prevention, interventional treatment techniques, and drug therapy, the long-term prognosis of patients with STEMI remains poor (1). Understanding the factors associated with mortality after STEMI can guide management decisions and provide valuable prognostic information for clinicians, patients, and their families. Currently, the most widely used risk prediction model for acute myocardial infarction (AMI) is the GRACE score and TIMI risk model (2). However, these models were developed before the widespread application of modern treatments for AMI, raising concerns about their predictive utility in current practice (3). Therefore, it is necessary to develop a new predictive model based on existing treatment techniques to better guide the management of myocardial infarction.

Skeletal muscle is the most common type of muscle in the body. It plays a critical role in physical function and metabolic health. The loss of skeletal muscle mass and function with age, also referred to as sarcopenia, has a major impact on quality of life and is associated with multiple adverse outcomes, such as physical frailty, all-cause mortality and increased healthcare costs. Previous studies have frequently revealed a strong association between low muscle mass and an increased risk of cardiovascular disease (4, 5). Low muscle mass is an independent risk factor for cardiovascular disease-related mortality (6). The infiltration of ectopic adipose tissue into skeletal muscle has become a focus of attention in the last 10 years. Myosteatosis is characterized by the ectopic deposition of adipose tissue between muscle groups (intermuscular fat) and within individual muscle fibers (intramuscular fat), which increases with age and is considered negatively correlated with muscle mass, mobility, strength, and metabolism (7). MMA is a measurement method for evaluating the average density or brightness of muscle tissue (8). There is increasing evidence that in cardiovascular diseases, myosteatosis may be more closely related to morbidity and mortality than muscle mass or size (9, 10).Emerging evidence supports the prognostic relevance of myosteatosis in various clinical settings, including coronary artery disease. For instance, recent research has demonstrated that skeletal muscle adiposity is closely related to coronary microvascular dysfunction and adverse cardiovascular outcomes (11). Additionally, abdominal CT-derived body composition thresholds using AI-based tools have been shown to predict long-term adverse outcomes with high accuracy (12). These findings reinforce the concept that myosteatosis is a critical factor in determining cardiovascular prognosis. However, the relationship between myosteatosis and STEMI has not yet been elucidated.

The aim of this study was to investigate the prognostic value of myosteatosis, assessed via MMA, in predicting long-term outcomes in patients with STEMI who underwent emergency PCI. We hypothesized that higher levels of myosteatosis, indicated by lower MMA values, were associated with poorer survival outcomes in these patients and that integrating myosteatosis with other clinical parameters could enhance the accuracy of prognostic prediction models for STEMI. Ultimately, we developed a myosteatosis-related nomogram model for predicting long-term prognosis in acute myocardial infarction patients.

## 2 Materials and methods

### 2.1 Patient population

This was a single-center retrospective cohort study. STEMI patients who had undergone PCI at the Heart Center of the First Affiliated Hospital of Lanzhou University in China from February 13, 2017, to December 31, 2020 were enrolled in this study. The inclusion criterion was a diagnosis of STEMI in accordance with ACC/AHA guidelines (13). All patients needed to undergo chest CT on the day of admission or soon after admission. The exclusion criteria were as follows: (1) treatment with a coronary artery bypass graft; (2) cerebral hemorrhage less than 1 year prior or cerebral infarction <6 months prior; (3) severe liver or kidney dysfunction; (4) tumor; (5) coagulopathy; (6) contraindication to antiplatelet drug treatment; (7) missing key clinical parameters necessary for prognostic analysis (e.g., creatinine, complete blood count, and troponin levels); (8) loss to follow-up; and (9) no chest computed tomography (CT) during hospitalization. After excluding patients who met the abovementioned exclusion criteria, 324 patients were ultimately included.

Our study protocol was in adherence to the ethical standards of the institutional research committees and the 1975 Declaration of Helsinki. The study protocol was approved by the ethics committee of the First Affiliated Hospital of Lanzhou University (No. LDYYLL-2024-796). The institutional review board waived the requirement for informed consent owing to the retrospective nature of the study.

### 2.2 Data collection

Demographic and angiographic data were obtained from the registry system of our hospital. Patient data, including age, sex, height, weight, systolic BP, diastolic BP, HR, LVEF, time from symptom onset to FMC, hypertension status, diabetes status, current smoking status, history of PCI, history of MI, history of AF, bleeding, acute heart failure, malignant arrhythmia, TnI level, creatinine level, hemoglobin (Hb) level and TIMI flow grade flow, were collected.

A 12-lead surface ECG was recorded upon admission. Echocardiography, performed by a cardiologist using Vivid 7 or Vivid E9 (GE Healthcare, Marlborough, MA, USA), assessed LVEF via the Simpson method upon admission, with transthoracic echocardiography completed within 24 hours post-PCI. Routine chest CT scanning was performed within 72 hours of admission on a Siemens SOMATOM Sensation 64-slice scanner, with patients positioned arms raised above head, excluding external foreign bodies, and instructed to hold their breath during the scan.

Bleeding was defined as bleeding academic research consortium (BARC) classification 3 or 5 bleeding. Malignant arrhythmias were defined as new-onset arrhythmias, including ventricular tachycardia (VT) and ventricular fibrillation (VF). Atrial fibrillation (AF) occurred during hospitalization.

### 2.3 CT data acquisition

**Figure 1:**
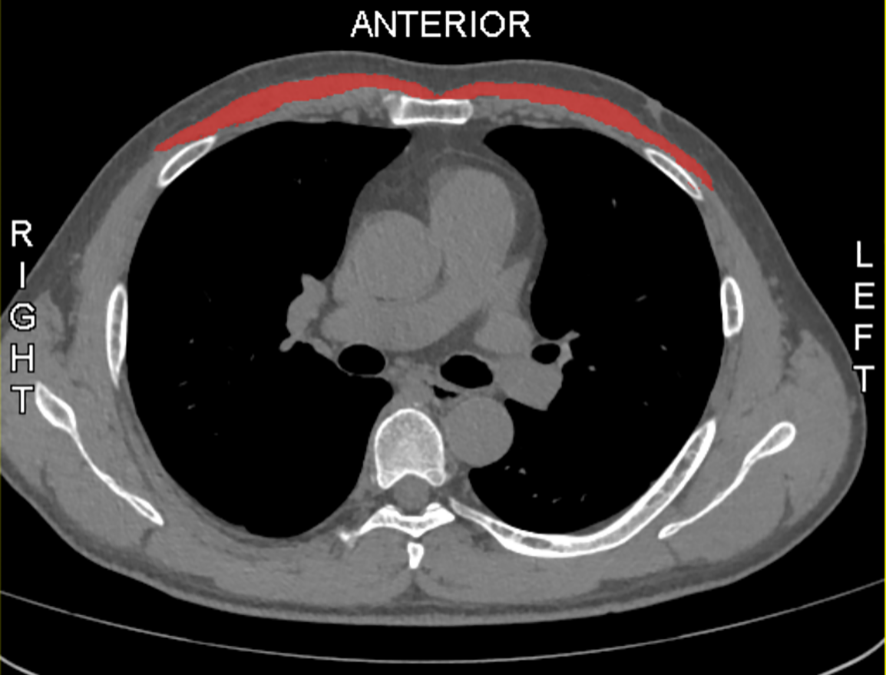
Sample axial CT images of the sixth thoracic vertebral region. Pectoralis muscles are in red.

The MMA was calculated using Slice-O-Matic software (version 5.0; Tomovision, Montreal, Canada) at the region of interest (ROI), defined by identifying the lower edge of the sixth thoracic vertebra (T6) in the sagittal plane and corresponding to the axial plane at the level of the fifth anterior rib, where the pectoralis major area, as the main muscle area, was manually outlined bilaterally (14). (**Figure 1**). The DICOM format of the selected chest CT slice was output and imported into software for image processing with an average filter (radius of 2 pixels), followed by initial segmentation in Threshold mode with HU ranges set (-29 to +150 HU) for skeletal muscle (15, 16). Boundaries were refined using the Region Growing module (seed point selected at the center, gradient threshold 5-10), followed by noise processing with the Morpho module (dilation and erosion each performed once, radius 1 pixel). Manual correction (Zoom In 2-4x, brush size 1-10 pixels) was applied, and TAG files were saved. Finally, Finally, the 2D result module was used to calculate the area of the pectoralis major muscle and the average HU value of this area, which is known as mean muscle attendance. All parameters were verified by two individuals to ensure segmentation accuracy.

### 2.4 Hospital−based follow−up

Specially trained nurses were responsible for follow-up under the supervision of cardiologists. Follow-up examinations were conducted immediately after the first consultation during hospitalization, at one month, three months, and six months after hospitalization, followed by annual assessments thereafter until the last recorded follow-up on April 24, 2023. Prognostic outcomes were tracked and recorded through outpatient visits, hospital admissions, and telephone follow-ups, with data on death, cause of death, and hospital admissions systematically documented during each consecutive consultation. The clinical endpoint was overall survival (OS), calculated between the time of surgery initiation, and mortality or the last follow-up occasion.

### 2.5 Statistical analysis

All the statistical analyses were performed using R (version 4.3.0 (2023-04-21 ucrt)). Continuous variables are presented as means ± SDs or medians with interquartile ranges and were compared using Student’s t test or the Wilcoxon rank-sum test. For categorical variables, number of total and the corresponding percentages were given and were compared using the chi-square test. Potential correlations between measured parameters and all-cause death were investigated through univariate and multivariate Cox proportional hazards models. AMI patients were divided into high- and low-risk groups according to the MMA score. A Kaplan-Meier survival curve was generated to compare OS between patients in the high- and low-risk groups. A nomogram for predicting the 1-, 3-, and 5-year OS of patients with AMI was constructed. The calibration curves and C-index were used to assess the discrimination ability of the nomogram. A p value <0.05 indicated significance.

## 3 Results

### 3.1 Baseline characteristics

A total of 324 patients (83.3% male) were ultimately eligible for this study; 35 of whom (10.8%) died during the follow-up period. **Table 1** shows all patient characteristics and the percentages of patients who died and did not die during the follow-up period. The mean ages of the patients who died and did not die during the follow-up period were 60.00 ± 11.28 years and 67.51 ± 9.53 years, respectively. A lower height and weight, longer time from symptom onset to FMC, and a greater proportion of patients requiring mechanical circulatory support. High incidences of bleeding, acute heart failure, malignant arrhythmia, newly diagnosed atrial fibrillation (AF), lower LVEF(<50%) and BP(SBP<90mmHg,DBP<60mmHg), and higher HR(>85bpm), serum creatinine(>108umol/L) and lower MMA(<32.5) were observed in patients who died after discharge (**Table 1**).

**Table 1:** Subject demographics.

		Survival Group	Mortality Group	*p*
		(n=289)	(n=35)	
Sex (%)	Male	247 (85.5)	23 (65.7)	0.007
	Female	42 (14.5)	12 (34.3)	
Age (mean (SD))		60.00 (11.28)	67.51 (9.53)	<0.001
Height (mean (SD))		168.78 (7.06)	165.26 (8.19)	0.007
Weight (mean (SD))		70.64 (11.28)	64.80 (13.65)	0.005
Symptom onset to FMC (%)	<6h	130 (45.0)	7 (20.0)	0.016
	6-12h	95 (32.9)	18 (51.4)	
	>12h	64 (22.1)	10 (28.6)	
TIMI grade flow (%)	0	137 (47.4)	21 (60.0)	0.49
	1	13 (4.5)	2 (5.7)	
	2	14 (4.8)	1 (2.9)	
	3	125 (43.3)	11 (31.4)	
MCS (%)	No	245 (84.8)	21 (60.0)	0.001
	Yes	44 (15.2)	14 (40.0)	
Hypertension (%)	No	162 (56.1)	12 (34.3)	0.024
	Yes	127 (43.9)	23 (65.7)	
Diabetes (%)	No	222 (76.8)	24 (68.6)	0.385
	Yes	67 (23.2)	11 (31.4)	
Current smoker (%)	No	157 (54.3)	19 (54.3)	1
	Yes	132 (45.7)	16 (45.7)	
History of PCI (%)	No	268 (92.7)	32 (91.4)	1
	Yes	21 (7.3)	3 (8.6)	
History of MI (%)	No	263 (91.0)	29 (82.9)	0.22
	Yes	26 (9.0)	6 (17.1)	
History of AF (%)	No	286 (99.0)	33 (94.3)	0.163
	Yes	3 (1.0)	2 (5.7)	
Bleeding (%)	No	280 (96.9)	27 (77.1)	<0.001
	Yes	9 (3.1)	8 (22.9)	
Acute heart failure (%)	No	278 (96.2)	30 (85.7)	0.022
	Yes	11 (3.8)	5 (14.3)	
Malignant arrhythmia (%)	No	285 (98.6)	29 (82.9)	<0.001
	Yes	4 (1.4)	6 (17.1)	
AF (%)	No	275 (95.2)	26 (74.3)	<0.001
	Yes	14 (4.8)	9 (25.7)	
Age groups (%)	<=65	190 (65.7)	13 (37.1)	0.002
	65+	99 (34.3)	22 (62.9)	
SBP (%)	<=90	14 (4.8)	5 (14.3)	0.062
	90+	275 (95.2)	30 (85.7)	
DBP (%)	<=60	32 (11.1)	10 (28.6)	0.008
	60+	257 (88.9)	25 (71.4)	
Heart rate (%)	<=85	193 (66.8)	14 (40.0)	0.003
	85+	96 (33.2)	21 (60.0)	
LVEF (%)	<50	115 (39.8)	24 (68.6)	0.002
	>=50	174 (60.2)	11 (31.4)	
Tnl (%)	<=1	116 (40.1)	13 (37.1)	0.874
	>1	173 (59.9)	22 (62.9)	
Creatinine (%)	<=108	272 (94.1)	27 (77.1)	0.001
	108+	17 (5.9)	8 (22.9)	
ANC (%)	<=12	243 (84.1)	27 (77.1)	0.423
	12+	46 (15.9)	8 (22.9)	
Hb (%)	<120	15 (5.2)	3 (8.6)	0.664
	>=120	274 (94.8)	32 (91.4)	
MMA(%)	<32.5	92 (31.8)	24 (68.6)	<0.001
	>=32.5	197 (68.2)	11 (31.4)	

FMC, first medical contact; MCS, mechanical circulatory support; PCI, percutaneous coronary intervention; MI, myocardial infarction; AF, atrial fibrillation; SBP, systolic blood pressure; DBP, diastolic blood pressure; LVEF, left ventricular ejection fraction; ANC, absolute neutrophil count; Hb, hemoglobin; MMA, mean muscle attenuation.

### 3.2 Identification of all-cause mortality-related indicators

In the univariable analysis, age (<65 years vs. ≥65 years, *p*<0.05), sex (female vs. male, *p*<0.05), time from symptom onset to FMC (6-12 h vs. <6 h, *p*<0.05), SBP(<90 mmHg vs. ≥90 mmHg, *p*<0.01), DBP(<60 mmHg vs. ≥60 mmHg, *p*<0.01), HR(<85 bpm vs. ≥85 bpm, *p*<0.01), LVEF(<50% vs. ≥50%, *p*<0.01),MCS (yes vs. no, *p*<0.01), history of hypertension (yes vs. no, *p*<0.05), history of AF (yes vs. no, *p*<0.05), bleeding (yes vs. no, *p*<0.01), acute heart failure (yes vs. no, *p*<0.05), malignant arrhythmia (yes vs. no, *p*<0.01), newly diagnosed AF (yes vs. no, *p*<0.01), creatinine (<108 umol/L vs. ≥108 umol/L, *p*<0.01), MMA (<32.5 vs. ≥32.5, *p*<0.01) were identified as significant prognostic factors of all-cause mortality. In the multivariable analysis, time from symptom onset to FMC (6–12 h vs. <6 h, *p*<0.05), SBP (<90 mmHg vs. ≥90 mmHg, *p*<0.01), DBP (<60 mmHg vs. ≥60 mmHg, *p*<0.05), HR (<85 bpm vs. ≥85 bpm, *p*<0.01), LVEF (<50% vs. ≥50%, *p*<0.01), MCS (yes vs. no, *p*<0.01), bleeding (yes vs. no, *p*<0.01), acute heart failure (yes vs. no, *p*<0.01), malignant arrhythmia (yes vs. no, *p*<0.01), newly diagnosed AF (yes vs. no, *p*<0.01), creatinine (<108 µmol/L vs. ≥108 µmol/L, *p*<0.05), and MMA (<32.5 vs. ≥32.5, *p*<0.01) were statistically significantly associated with all-cause mortality in patients with STEMI (**Table 2**).

**Table 2:** Univariate and multivariate regression analysis of all-cause mortality-related indicators for STEMI.

		Univariable analysis		Multivariable analysis^a^	
		HR (95%CI)	*p*	HR (95%CI)	*p*
Sex	Female	2.42 (1.2-4.91)	0.014		
	Male	1			
Age (years)	≥65	2.73 (1.37-5.44)	0.0044		
	<65	1			
Height		0.95 (0.91-0.99)	0.0222	0.98 (0.92-1.04)	0.4729
Weight		0.96 (0.93-0.99)	0.0058	0.98 (0.94-1.01)	0.1639
Symptom onset to FMC	6-12h	2.83 (1.18-6.79)	0.0197	2.65 (1.1-6.36)	0.0294
	<6h	1		1	
TIMI grade flow	1	0.93 (0.22-4.03)	0.9275		
	2	0.63 (0.08-4.67)	0.6384		
	3	1			
MCS	yes	3.19 (1.61-6.32)	0.0009	3.45 (1.72-6.89)	0.0005
	no	1		1	
Hypertension	yes	2.07 (1.03-4.18)	0.0408	1.65 (0.8-3.38)	0.175
	no	1		1	
Diabetes	yes	1.25 (0.6-2.59)	0.5505		
	no	1			
Current smoker	yes	0.95 (0.49-1.86)	0.8783		
	no	1			
History of PCI	yes	1.48 (0.45-4.85)	0.518		
	no	1			
History of MI	yes	2.14 (0.88-5.18)	0.0926		
	no	1			
History of AF	yes	4.21 (1.01-17.61)	0.0492	2.76 (0.64-11.83)	0.1718
	no	1		1	
Bleeding	yes	6.22 (2.78-13.92)	0	5.23 (2.3-11.89)	0.0001
	no	1		1	
Acute heart failure	yes	3.21 (1.23-8.36)	0.0173	4.03 (1.52-10.66)	0.005
	no	1		1	
Malignant arrhythmia	yes	8.99 (3.68-21.97)	0	7.78 (3.13-19.31)	0
	no	1		1	
Newly diagnosed AF	yes	4.08 (1.87-8.9)	0.0004	3.63 (1.65-7.96)	0.0013
	no	1		1	
SBP (mmHg)	≥90	0.28 (0.11-0.73)	0.0094	0.24 (0.09-0.63)	0.0036
	<90	1		1	
DBP (mmHg)	≥60	0.3 (0.15-0.64)	0.0017	0.38 (0.18-0.8)	0.0106
	<60	1		1	
Heart rate (bpm)	≥85	2.73 (1.39-5.38)	0.0036	2.85 (1.44-5.64)	0.0027
	<85	1		1	
LVEF (%)	≥50	0.36 (0.17-0.73)	0.005	0.35 (0.17-0.72)	0.0042
	<50	1		1	
TnI (ng/ml)	≥1	1.11 (0.56-2.21)	0.76		
	<1				
Creatinine (umol/L)	≥108	3.8 (1.71-8.43)	0.001	2.93 (1.29-6.67)	0.0102
	<108	1		1	
ANC (10^9^/L)	≥12	1.31 (0.59-2.9)	0.5016		
	<12	1			
Hb (g/L)	≥120	0.62 (0.19-2.03)	0.4284		
	<120	1			
MMA (Hu)	≥32.5	0.24 (0.12-0.5)	0.0001	0.3 (0.13-0.7)	0.0049
	<32.5	1		1	

FMC, first medical contact; MCS, mechanical circulatory support; AF, atrial fibrillation; SBP, systolic blood pressure; DBP, diastolic blood pressure; LVEF, left ventricular ejection fraction; MMA, mean muscle attenuation.

a Adjusted for age and sex.

### 3.3 Prognostic value of the MMA

**Figure 2:**
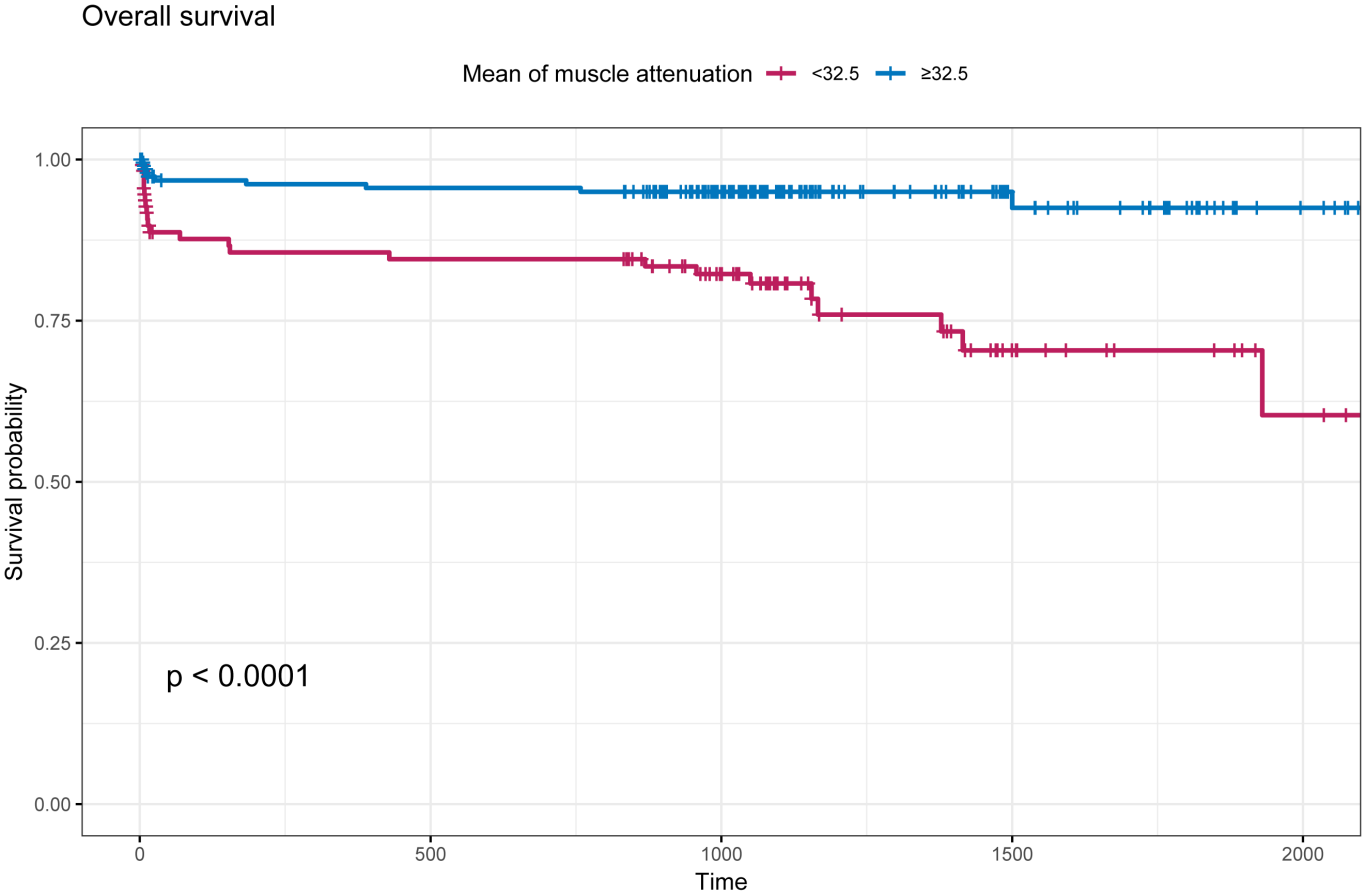
Kaplan-Meier survival curve based on mean muscle attenuation (MMA). The cut-off values defining the MMA was 32.5 Hu. Blue line indicates patients with MMA≥32.5 Hu; Red line indicates patients with MMA<32.5 Hu.

The intra-group correlation coefficient for MMA values assessed by two independent observers blinded was 0.938 (P<0.001, 95% CI [0.923-0.950]).The optimal cutoff MMA value for the prediction of all-cause mortality was 32.5 Hu. The patients were classified into high (≥32.5, n=208) and low (<32.5, n=116) MMA groups according to the MMA value. As shown in (**Figure 2**), the survival time of the patients in the low-MMA group was shorter than that of those in the high-MMA group.

### 3.4 Construction of the prognostic nomogram

**Figure 3:**
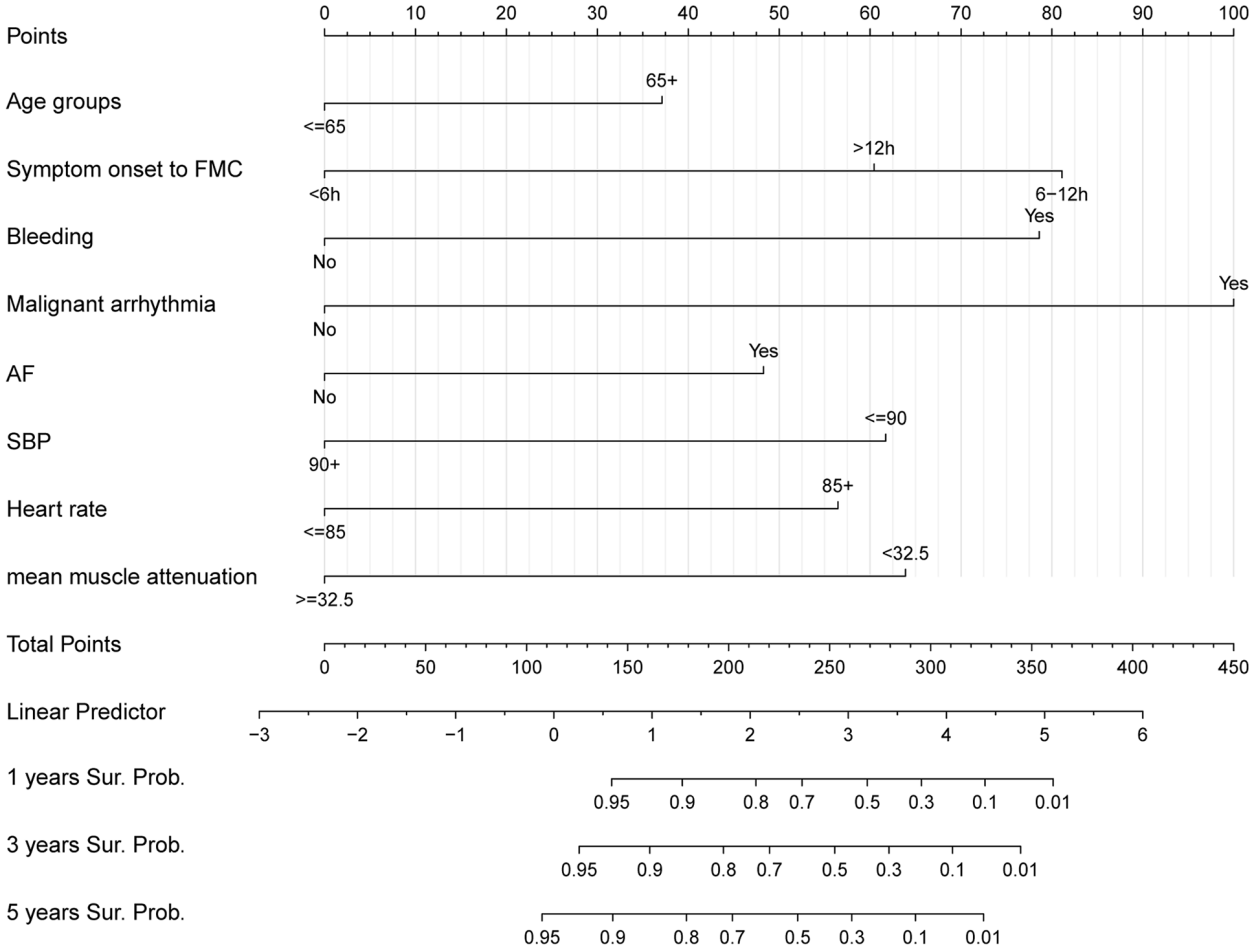
Nomogram for predicting the 1,3,5-year survival in patients with ST-elevation myocardial infarction.

**Figure 4:**
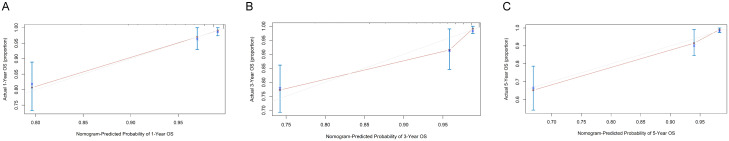
Calibration curve of the nomogram. **(A)** Calibration analysis to predict 1-year overall survival (OS); **(B)** Calibration analysis to predict 3-year OS; **(C)** Calibration analysis to predict 5-year OS.

We created a nomogram with the C-index equal to 0.86 integrating MMA and other clinical parameters to evaluate the long-term prognosis of patients with STEMI (**Figure 3**). Calibration analyses indicated a stable consistency between the predicted and actual survival rates at 1, 3, and 5 years (**Figure 4**).

## 4 Discussion

Our study revealed a significant connection between myosteatosis and a poor prognosis in patients with STEMI. When the studied patients were classified by a MMA of 32.5, the patients with a high MMA had significantly longer OS than those with low MMA. Multivariate Cox regression analysis revealed that MMA<32.5 was an independent risk factor for OS. Moreover, we constructed a prediction model that integrates the MMA and other clinical parameters.

### 4.1 Myosteatosis and STEMI

In recent years, the assessment of skeletal muscle function has been increasingly recognized in health management. This is because an increasing number of studies have shown that the functional status of skeletal muscles is closely related to disease outcomes, such as sarcopenia (17, 18). In 2018, the European Working Group on Sarcopenia in Older People (EWGSOP) updated the original definition of sarcopenia and noted that muscle quality is as important as muscle quantity (19). Many studies have explored the relationships between AMI and muscle quantity parameters, including the appendicular skeletal muscle index (ASMI) with the visceral or subcutaneous (V/S) fat ratio (20), skeletal muscle mass (SMM) (21), and skeletal muscle cross-sectional area (SMA) (22). However, muscle quality has been less explored than muscle mass. Muscle quality is closely associated with fatty infiltration of the muscle (i.e., myosteatosis). Like muscle mass, myosteatosis is associated with cardiovascular disease and has been recognized as an important concept in the field of sarcopenia (23). However, the clinical significance of the MMA in patients with STEMI has not been elucidated. Our study revealed that the risk of future adverse events was significantly higher in STEMI patients with a low MMA than in those with a high MMA. This observation is partly supported by the study by Yamashita M., who analyzed the correlation of MMA with prognosis in patients with cardiovascular disease who underwent surgery (11, 24). Additionally, there is currently no standardized cutoff value for the diagnosis of myosteatosis (25). Our study revealed that the optimal MMA cutoff value for predicting the prognosis of STEMI patients was 32.5, which is close to the cutoff values reported in many previous studies (26).

### 4.2 The mechanism of the impact of myosteatosis on clinical prognosis

The mechanism of the relationship between myosteatosis and mortality has not been well explored. However, metabolic and mechanical dysfunction of skeletal muscles may be a possible explanation. Myosteatosis can lead to a chronic inflammation state, which increases insulin resistance. Insulin resistance can lead to lipid abnormalities, such as reduced high-density lipoprotein cholesterol, which contribute to the development of atherosclerosis. Additionally, insulin resistance is closely associated with chronic low-grade inflammation and oxidative stress, all of which accelerate the development of cardiovascular disease (27–29). On the other hand, myosteatosis leads to the conversion of type II muscle fibers into type I fibers, which may affect the strength of muscle contraction and speed abilities, reduce cardiovascular function, and ultimately lead to weakness and death (30).

### 4.3 A novel risk predictive model for STEMI based on myosteatosis

Currently, the major risk prediction models for STEMI include the TIMI risk score, PAMI score, Zwolle risk score, CADILLAC risk score, and GRACE score. However, these risk scores were developed before the widespread use of contemporary AMI therapies, which raises concerns about their predictive utility in current practice (2).Therefore, we created a novel risk predictive model for STEMI on the basis of age, time from symptom onset to FMC, bleeding, malignant arrhythmias, atrial fibrillation, lower baseline systolic blood pressure, increased heart rate and MMA. The present results suggest that the risk model performs well in predicting the OS of STEMI patients. Previous studies have revealed that advanced age, prolonged time from symptom onset to FMC, malignant arrhythmias, atrial fibrillation, lower baseline systolic blood pressure, increased heart rate during the episode, and bleeding contribute to an increased mortality rate in AMI patients (31–34). Similar to these studies, our study revealed that these factors were associated with an increased risk of all-cause mortality. This evidence further enhances the reliability of our model. In addition, this model uses recent clinical data, which is in line with existing methods for clinical diagnosis and treatment. Moreover, the indicators included in the risk model are common clinical data and easy to obtain, making them suitable for widespread clinical applications. In summary, the identified risk model is a practical tool to help making clinical decisions.

### 4.4 Limitation

Our study has several limitations. First, it was a single-center retrospective cohort study with a small sample of patients; such studies are prone to biases. Second, the study was limited to STEMI patients, thus potentially limiting the generalizability of the findings. Third, physical activity tests, such as grip strength tests and walk tests, were not performed despite their importance for assessing muscle quality (35). To increase the robustness and generalizability of our conclusions, large-sample, multicenter prospective studies involving diverse malignancies are warranted.

## 5 Conclusion

In conclusion, the results indicate that MMA-defined myosteatosis may serve as an independent prognostic factor for OS in patients with STEMI. Therefore, the assessment of myosteatosis in addition to muscle mass is expected to help better assess the prognosis of AMI patients and guide clinical decision-making. Additionally, in this study, we established a novel risk model comminating myosteatosis with other common clinical indicators and reported the high predictive efficacy of the model for the prognosis of STEMI patients. These findings provide valuable insights into the prognostic landscape of STEMI.

## Data Availability

The original contributions presented in the study are included in the article/supplementary material. Further inquiries can be directed to the corresponding author.
